# Advances in sarcopenia and urologic disorders

**DOI:** 10.3389/fnut.2024.1475977

**Published:** 2024-11-06

**Authors:** Tonglei Zhao, Weipu Mao, Mingjin Hu, Qingyang Yu, Xinyang Peng, Jie Ji, Jianguo Qiu, Jianping Wu

**Affiliations:** ^1^Southeast University School of Medicine, Nanjing, China; ^2^Department of Urology, Affiliated Zhongda Hospital of Southeast University, Nanjing, China; ^3^Surgical Research Center, Institute of Urology, Southeast University Medical School, Nanjing, China; ^4^Department of Urology, Nanjing Lishui People’s Hospital, Zhongda Hospital Lishui Branch, Southeast University School of Medicine, Nanjing, China; ^5^Department of Urology, Lianshui People's Hospital of Kangda College Affiliated to Nanjing Medical University, Jiang Su, China

**Keywords:** sarcopenia, overactive bladder, polycystic kidney, bladder cancer, prostate cancer

## Abstract

Sarcopenia is a loss of muscle strength, muscle mass, and function that can increase a patient’s risk of injury, illness, and can even severely impair quality of life and increase a patient’s risk of death. A growing body of research suggests that sarcopenia and urinary tract disorders are closely related. In this review, we aimed to emphasize the definition of skeletal sarcopenia, summarize the methods used to diagnose skeletal sarcopenia, discuss the advances in the study of sarcopenia in benign diseases of the urinary system, discuss the advances in the study of sarcopenia in malignant diseases of the urinary system. Sarcopenia and urologic diseases interact with each other; urologic diseases cause sarcopenia, and sarcopenia aggravates the condition of the original disease, thus falling into a vicious circle. This review provides a comprehensive understanding of sarcopenia in urologic diseases, which is very important for the management and prognosis of urologic diseases.

## 1 Introduction

With the aggravation of the aging trend of the national population, sarcopenia has become a global public health problem, and the incidence of sarcopenia is increasing year by year (1). Sarcopenia is a progressive and systemic skeletal muscle disease that involves accelerated loss of muscle mass and function and is associated with increased adverse outcomes such as falls, decreased function, weakness, and mortality (2).

There is an interaction between sarcopenia and urinary tract disorders. Sarcopenia can lead to chronic inflammation, loss of muscle strength and etc. (3). Therefore, patients with sarcopenia have an increased chance of developing urinary tract diseases such as urinary tract inflammation and overactive bladder disease, and even aggravate the progression of diseases such as chronic kidney disease (CKD) and urological malignancies (4–7). Urologic malignancies lead to sarcopenia by causing a decrease in protein intake, an increase in protein catabolism, and a decrease in physical activity, which further affects aspects of surgery, chemotherapy, and prognosis (8, 9). Although studies in recent years have suggested that sarcopenia is associated with urologic disorders, little attention has been paid to the potential role of the urinary system in the etiology of sarcopenia and in interventions. Therefore, in this review, we aimed to (1) emphasize the definition of skeletal sarcopenia, (2) summarize the methods used to diagnose skeletal sarcopenia, (3) discuss the advances in the study of sarcopenia in benign and malignant diseases of the urinary system.

## 2 Sarcopenia

### 2.1 Definitions

The term sarcopenia was first coined in 1988 by Rosenberg, who argued that no single feature of age-related decline is more pronounced than weight loss, which affects walking, capacity intake, overall nutritional intake, and more. Thus, initially sarcopenia was considered an age-related loss of skeletal muscle mass (10).

Different definitions of sarcopenia have been proposed by various organizations to date, with the EWGSOP2 definition being the most widely used in clinical practice (11–16). In 2010, EWGSOP published a definition of sarcopenia that is widely used worldwide. Sarcopenia is defined as a disease of old age caused by a decrease in muscle mass, muscle strength and/or physical function (14). In 2018, EWGSOP2 updated and added to the definition of sarcopenia: “sarcopenia is a muscle disease (muscle failure) rooted in adverse muscle changes that accrue across a lifetime; sarcopenia is common among adults of older age but can also occur earlier in life.” In contrast to the 2010 definition, in the updated definition, EWGSOP2 includes low muscle strength as the main indicator of sarcopenia (12). Because an increasing number of scholars believe that muscle number and muscle mass remain problematic as the primary parameters for defining sarcopenia due to technical limitations, muscle strength is preferred over muscle mass in predicting poor outcomes, and muscle strength is currently the most reliable measure of muscle function (17–19). EWGSOP2 diagnostic thresholds are as follows: 1. Low muscle strength: Grip strength <27 kg for men and < 16 kg for women or Chair Rise Test: 5 rises in >15 s; 2. Low muscle mass: ASM (appendicular skeletal muscle mass)/height2 ≤ 7 kg/m2 for men and ≤ 5.5 kg/m2 for women; 3. Low physical performance: Step speed ≤0.8 m/s or SPPB (Simple Physical Performance Test) score ≤ 8 or TUG (timed up and go walking test) ≥20 s or 400 m walk test not completed or completed in ≥6 min (12).

Another widely used consensus on sarcopenia is AWGS (Asian Working Group for Sarcopenia), which in 2014 adopted a similar definition to EWGSOP, an age-related geriatric syndrome characterized by decreased muscle mass and low muscle function. The difference is that AWGS requires both low muscle strength and low physical performance to be diagnosed. Additionally, threshold values have been proposed that are compatible with Asian populations based on their anthropometrics (20). In the latest AWGS 2019 consensus, the diagnostic thresholds are as follows: 1. low muscle strength: grip strength <28 kg in men and < 18 kg in women; 2. low muscle mass: ASM/height^2^ < 7.0 kg/m^2^ for men and < 5.4 kg/m^2^ for women as measured by DXA (Dual-energy X-ray bone densitometry); <7.0 kg/m^2^ for men and < 5.7 kg/m^2^ for women as measured by BIA (bioelectrical impedance analysis); 3. low physical performance: gait speed <1.0 m/s or chair rise test ≥12 s or SPPB ≤9. AWGS 2019 also added recommendations for different algorithms to be used in community and hospital settings and introduced the term “possible sarcopenia” to promote early intervention in community settings. Possible sarcopenia is defined as low muscle strength (handgrip strength) or low physical performance (chair rise test) (21).

However, these criteria of weight loss as the main criterion for sarcopenia are not met in certain diseases, such as diabetes (22). In these disorders, the patient’s muscle mass decreases but fat mass remains the same or increases, a condition known as sarcopenic obesity (SO). The European Society for Clinical Nutrition and Metabolism (ESPEN) and the European Association for the Study of Obesity (EASO), as well as the jointly appointed international expert panel proposes that SO is defined as the co-existence of excess adiposity and low muscle mass/function (23).

### 2.2 Diagnosis

#### 2.2.1 Screening

Early screening and early intervention for skeletal sarcopenia is important. There are a number of clinical screening tools available to identify sarcopenia (Table 1). SARC-F is currently the most recommended screening tool for sarcopenia, but its sensitivity is low. Researchers have also developed/re-purposed several other screening tools for effective screening. Each of these screening tools has advantages and disadvantages for different clinical use scenarios. Screening tools do not serve as a definitive diagnosis of sarcopenia, and if a screening tool suggests the presence of sarcopenia, a definitive test should be performed.

**Table 1:** Screening tools for sarcopenia.

Name	Domains measured	Strengths	Limitations	Clinical scenarios
SARC-F	Strength Assistance in walking Rise from a chair Climb stairs Falls	Initial diagnosis of sarcopenia Quick and easy to use Suitable for all clinical scenarios High specificity and moderate sensitivity	The first four measures may be biased Muscle function was mainly assessed, and muscle mass was not assessed Low sensitivity	Suitable for virtually all medical environments
SARC-F Calf	Strength Assistance in walking Rise from a chair Climb stairs Falls Calf circumference	Increased sensitivity for SARC-F (45.9%-57.2%)	Calf measurements are susceptible to interference from other factors such as fat and edema	For community health, specialty clinical settings
Ishii Test	Age Grip strength Calf circumference	High sensitivity and specificity (84.9% and 88.2% for men; 75.5% and 92.0% for women)	Not yet validated in multiple populations	Not commonly used in primary care, generally used in specialty clinical settings
MSRA	MSRA-5 includes: Age Protein Physical activity level Number of hospitalizations Weight loss in the last year MSRA-7 includes: Age Protein Dairy products consumption Number of meals per day Physical activity level Number of hospitalizations Weight loss in the last year	High sensitivity	Not yet validated in multiple populations	Generally not used in community hospitals
SarSA-Mod	Age Weight Calf circumference	High sensitivity (84.3% in women, 85.4% in men)	Validated in Middle Eastern populations only	Used in a variety of clinical settings
Grip strength	Low muscle strength was defined as hand grip strength: < 30 kg in men < 20 kg in women	Easy to perform in clinic setting with simple, inexpensive tool dynamometer	Requires dynamometer	Widely used in clinical practice
SarQoL questionnaire	A scale consisting of 55 items in 7 domains in the form of 22 questions	Assisting health care providers in assessing patients' perceptions of aspects of their physical, mental and social health	The verification crowd is limited to French-speaking people Participants were mostly community females, and measurements may be biased	For clinical care and research
Fracture risk assessment tool	Age Sex Weight Height Previous fracture Parental hip fracture Glucocorticoid use Rheumatoid arthritis Smoking Alcohol consumption Secondary osteoporosis Bone mineral density with or without bone	The FRAX score without bone density can be calculated using an online calculator High sensitivity (90.9%)	Non-response bias may exist	
Taiwan Risk Score for Sarcopenia	Age Sex Underweight Receipt of social assistance pension Lack of exercise Fasting blood glucose Abnormal creatinine levels	High sensitivity (71.8%) High specificity (71.1%)	Long time for evaluation; Rarely used at present	Be applied cost-effectively in the community for early detection of sarcopenia.
Anthropometric prediction equation (PE)	Anthropometric equations constructed to predict skeletal muscle mass in the limbs based on weight, BMI, age, and sex. "ASM=10.05+0.35×Body weight-0.62×BMI-0.02×age+5.10 (male)"	High specificity (male: 99.5%; female: 94.7%)	Only been used in the community's older population;	Screening for sarcopenia in primary and aged care
Chair rise	Strength Cut-off points >15 s for 5 chair rises	Easy to perform in clinic setting Requires no specialized tools		

SARC-F, Strength, Assistance Walking, Rise from a Chair, Climb Stairs, and Falls; MRSA, Mini Sarcopenia Risk Assessment Questionnaire; SarSA-Mod, Sarcopenia Scoring Assessment Models; SarQoL, Sarcopenia Quality of Life; FRAX, Fracture Risk Assessment Tool; BMI, Body Mass Index.

#### 2.2.2 Physical performance and muscle strength tests

Physical performance is defined as visually measurable whole-body function related to exercise, involving not only muscles but also central and peripheral nerve function. There are several instruments that can be used for fitness testing, including gait speed, the Simple Physical Performance Test (SPPB), timed up and go walking test (TUG), and 400-meter walk test. Gait speed is now widely used in practice, with the commonly used gait speed test being the 4-meter walking speed test, and the EWGSOP2 recommending a speed of ≤0.8 m/s as an indicator of severe sarcopenia (24). The SPPB is a comprehensive test that incorporates a gait speed test, a balance test, and a chair stand test, and is scored out of 12, with a score of ≤8 indicating poor physical performance (25). In the TUG test, participants were asked to stand up from a standard chair, walk to a marker 3 meters away, turn around and walk back to sit in the chair, taking ≥20 s to be considered a poor physical performer. The 400-meter walk test assessed walking ability and endurance, in which participants were asked to complete 20 laps of a 20-meter walk as fast as possible for each lap, with two rest stops permitted during the test, and a final time of ≥6 min to be considered a poor performer. Considered to have poor physical performance (26).

Grip strength is easy and inexpensive to measure and can be used as a test tool for arm muscle strength. Grip strength correlates with strength in other parts of the body, so it can be a reliable alternative to more complex measurements of arm and leg strength. Accurate measurement of grip strength requires the use of a calibrated hand-held dynamometer under well-defined test conditions (27).

Seat Stand Test can be used as a test tool for leg muscle strength. It measures the time it takes for a patient to get up in a seated position five times without using their arms. Or it counts the number of times a patient can stand up and sit in a chair in a 30-s period of time (28).

#### 2.2.3 Imaging modalities and body composition measurement tools

Muscle mass can be reported in terms of whole-body skeletal muscle mass (SMM), appendicular skeletal muscle mass (ASM), or muscle cross-sectional area for specific muscle groups. Table 2 summarizes imaging measurement tools as well as body composition measurement tools.

**Table 2:** Imaging modalities and body composition measurement tools for sarcopenia.

Name	Strengths	Limitations	Clinical scenarios
L3-CT	L3 levels are strongly correlated with whole body muscle mass and fat mass	Not universally used Cannot control bias due to height error Limited availability Lack of portability High cost and radiation exposure	Suitable for cancer patients, seriously ill patients, liver disease patients
Mid-thigh imaging (MR/CT)	Considered the gold standard noninvasive tool for assessing muscle mass	Higher equipment costs Lack of portability Higher operator requirements	Not commonly used in primary care, generally used in specialist clinical settings
Muscle ultrasound	Portable Inexpensive Non-invasive No ionizing radiation Highly reproducible	Lack of harmonized diagnostic criteria	Clinical pilot studies
DXA	Provides ASM reproducibility estimates in minutes	Inconsistent results from different brands of machines Affected by patient hydration status	Generally not used in community hospitals
BIA	Low cost Portable	Lack of standardization limits its accuracy	Used in a variety of clinical environments

CT, Computed Tomography; MR, Magnetic Resonance; DXA, Dual-energy X-ray Absorptiometry; BIA, Bioelectric Impedance Analysis; ASM, Appendicular Skeletal Muscle Mass.

Magnetic resonance imaging (MRI) and computed tomography (CT) are considered the gold standard for noninvasive assessment of muscle mass (28). However these tools are not commonly used in primary care due to the high cost of the equipment, lack of convenience, and the need for a trained professional to use them. In addition, the cut-off point for measuring low muscle mass is not well defined. Studies have shown that CT images of specific lumbar spine markers correlate significantly with whole-body muscle. L3-CT imaging of the third lumbar spine is not limited to patients with cancer; this parameter has also been used as a predictor of mortality and other prognostic indicators in intensive care units and in patients affected by liver disease (29). Mid-thigh imaging (MRI or CT) is a good predictor of skeletal muscle mass throughout the body, and its correlation is stronger than the correlation between L1-L5 psoas muscle area and total body muscle (29).

Dual-energy X-ray bone densitometry (DXA) is a more widely available instrument for non-invasive determination of muscle mass. The advantage of the DXA is that it provides a reproducible estimation of ASM within a few minutes when using the same instrument and cut-off points. However, DXA instruments are not yet portable for use in the community, which is a disadvantage (30).

Bioelectrical impedance analysis (BIA) has been used to estimate SMM or ASM, BIA devices do not measure muscle mass directly but estimate muscle mass based on whole body conductivity. BIA requires specific conversion equations for calibration. For example, the Sergi equation is based on older European populations, and other relevant differences such as age and ethnicity of these populations and patients should be considered in clinical work, in addition to the fact that BIA measurements are affected by the hydration status of the patient. However, BIA needs more studies to validate population-specific prediction equations (31).

Muscle ultrasound assessment of pterygoid muscles such as the quadriceps can detect reductions in muscle thickness and cross-sectional area in a relatively short period of time, and therefore the technique has great potential for clinical application. The EuGMS Myasthenia Gravis Group has recently proposed a consensus protocol for muscle assessment using ultrasound, which involves the measurement of muscle thickness, cross-sectional area, fascicle length, droop angle, and echogenicity. Echogenicity reflects muscle mass because the non-contractile tissue associated with myasthenia gravis is highly echogenic (32). Therefore, ultrasound has the advantage of being able to assess muscle quantity and quality. Echo intensity is shown to be more useful than traditional sarcopenia-related parameters in predicting hospital-related complications in older acutely hospitalized patients (33).

Skeletal Muscle mass Index (SMI) is an indicator for assessing muscle mass, which is calculated by the formula ASM/height2 (34), so the measurement of SMI usually relies on the aforementioned imaging techniques, such as dual-energy x-ray absorptiometry (DXA), computed tomography (CT), and magnetic resonance imaging (MRI). As a superior index of sarcopenia, SMI not only predicts long-term survival in patients with urologic cancers, but also provides guidance on discharge management after surgery (35, 36). In addition, with advances in imaging technology, automated muscle segmentation and analysis methods are being developed to improve the accuracy and reproducibility of SMI measurements (37, 38). These studies contribute to a better understanding and application of SMI to improve the diagnosis and management of sarcopenia.

#### 2.2.4 Laboratory biomarkers

Creatine is produced by the liver and kidneys and taken up by myocytes, a portion of which is irreversibly converted to creatinine phosphate on a daily basis, and excess creatine in the circulation is converted to creatinine and excreted in the urine (39). Creatinine excretion rate is a promising metabolic index for assessing whole-body muscle mass. Fasting patients were orally administered an appropriate dose of deuterium-labeled creatine, followed by determination of labeled and unlabeled creatine and creatinine in urine by liquid chromatography and tandem mass spectrometry (40). Study Shows Creatine Dilution Test Results Correlate Well with MRI-Based Measures of Muscle Mass and Moderately with BIA and DXA Measures (41, 42). The test currently requires a significant amount of research to provide further improvements.

Potential biomarkers may also include markers of neuromuscular junctions, muscle protein transitions, behaviorally mediated pathways, inflammation-mediated pathways, redox-related factors and hormones or other anabolic factors (43). However, due to the complex pathophysiology of sarcopenia, there is still no single biomarker to recognize this condition in heterogeneous populations of young and old.

## 3 Sarcopenia and benign diseases of the urinary system

### 3.1 Inflammation of the urinary system

Inflammatory cytokines have been shown to promote muscle wasting, stimulate protein metabolism and inhibit protein synthesis (44, 45). Among many inflammatory cytokines, the main ones are tumor necrosis factor-*α* (TNF-α), C-reactive protein (CRP), interleukin (IL)-6. TNF-α is a key stimulator of chronic inflammation that triggers skeletal muscle contractile dysfunction. TNF-α inhibits protein synthesis via the IGF1/AKT pathway, thereby triggering muscle atrophy (46, 47). Sarcopenia was strongly associated with serum CRP levels, an indicator of infection (*p* < 0.0001) (48). Sustained elevation of IL-6 aggravates muscle atrophy by reducing muscle anabolism and damaging energy homeostasis, and it also directly mediates muscle catabolism (49). Increased IL-6 stimulates muscle protein degradation by interfering with insulin/PI3K/AKT signaling (50, 51).

Urinary tract infections are a common infectious disease that can occur anywhere in the urinary tract. In addition to simple urinary tract infections that can resolve on their own or be treated with antibiotics, there are more complex forms such as catheter-associated urinary tract infections. Catheter-associated urinary tract infections are also a common complication in trauma hospitalized patients, and a study by James DeAndrade et al. showed that myasthenia gravis was an independent risk factor for catheter-associated urinary tract infections (*p* = 0.011) (52).

### 3.2 Overactive bladder

Overactive bladder (OAB) is a syndrome characterized by symptoms of urinary urgency, typically accompanied by urinary frequency and nocturia, and may or may not involve urge incontinence. It is not associated with urinary tract infections or other definitive pathological changes. Recent studies have explored the relationship between sarcopenia and OAB, with promising findings.

A retrospective study conducted by Song et al. demonstrated a positive association between sarcopenia and the risk of OAB in adults in the United States (53). The study also suggested that sarcopenia could serve as a predictor for OAB. Similarly, Ida S et al. conducted a cross-sectional study which revealed a significant association between sarcopenia and OAB in elderly male patients with diabetes (5). Furthermore, Hashimoto et al. identified sarcopenia and visceral fat accumulation as potential risk factors for severe storage symptoms in female patients aged 65 years and older (54). While these studies suggest a possible link between sarcopenia and OAB, there is a need for further research and validation using multi-center data and larger sample sizes. Additionally, the underlying mechanism by which sarcopenia contributes to the development of OAB remains unknown.

In conclusion, sarcopenia may be a risk factor for developing OAB, but more research is needed to establish a definitive relationship. Further studies should aim to explore the mechanisms underlying this association and confirm the findings using larger and more diverse study populations.

### 3.3 Polycystic kidney

Polycystic kidney disease (PKD) is a benign urologic disorder of genetic origin. While generally non-malignant, it can still harm kidney function and necessitate dialysis treatment in extreme circumstances. PKD patients typically have numerous cysts, and their body mass index (BMI) may conceal underlying underweight issues. Consequently, Chih-Horng Wu and his team employed total abdominal muscle area (TAM) at the third lumbar vertebrae as a diagnostic standard for sarcopenia. Their research concluded that sarcopenia can be accurately diagnosed in PKD patients using CT and MRI scans (55). However, the disease may be hidden by the cysts. There was a negative correlation between kidney volume and abdominal muscle mass, but not with adipose tissue. Additionally, factors such as age, BMI, serum creatinine levels, and kidney volume play a crucial role in muscle loss in PKD patients.

A study by Lee et al. indicated that good nutritional status helps preserve renal function in PKD patients (56). Analysis from a two-year randomized controlled trial, CRAD001ADE12, suggests that the accelerated growth of cysts in patients with autosomal dominant polycystic kidney disease (ADPKD) can be slowed down with the use of everolimus, a mammalian target of rapamycin (mTOR) inhibitor (57). However, this substance is also associated with weight loss, particularly in women. This impact is possibly due to a decrease in food intake, including fat and protein, induced by the central nervous system, along with an increase in fat oxidation and mobilization. In skeletal muscles, glucose uptake and oxidation might be decreased, potentially leading to cachexia and muscle atrophy. Such findings are of significant importance for PKD patients undergoing immunosuppressive mTOR inhibitory therapy.

Ryu et al. demonstrated in a cross-sectional study that adopting the Dietary Approaches to Stop Hypertension (DASH) dietary pattern can help protect muscle strength in PKD patients. Therefore, a DASH diet might be effective in maintaining muscle strength and preventing sarcopenia among patients with ADPKD (58).

### 3.4 Kidney stone

Kidney stone disease is one of the most common benign diseases of the urinary system. A recent study points to a strong correlation between kidney stones and sarcopenia and that sarcopenia is an independent risk factor for kidney stones (59). Another study also noted that the risk of developing kidney stones decreased with an increase in the muscle-fat ratio (60). However, more research is needed to confirm these studies as they were all cross-sectional studies from the same database and did not look more closely at the relationship between stone composition, stone location, stone size and sarcopenia.

## 4 Sarcopenia and malignant diseases of the urinary system

### 4.1 Bladder cancer

Bladder cancer is one of the most common malignant tumors of the urinary system. Worldwide, the incidence of bladder cancer ranks 10th among malignant tumors, and the incidence in men is four times higher than that in women (61). Bladder cancer is categorized into non-muscle-invasive (NMIBC) and muscle-invasive (MIBC) (62). These two subtypes are treated differently and have different prognoses. Treatment of NMIBC includes transurethral cystectomy of the bladder tumor and postoperative intravesical instillation of chemotherapeutic agents or immunotherapy (63). Radical cystectomy and pelvic lymphadenectomy are considered the gold standard for the treatment of MIBC and high-grade NMIBC, which may be followed by chemotherapy or neoadjuvant chemotherapy (64).

Mechanistically, BCa has been shown to induce an inflammatory microenvironment through the release of cytokines including TNF-*α* (65, 66). Up-regulation of the ATP-ubiquitin-protein pathway may also protein degradation and tissue wasting. In addition, animal models have demonstrated that BCa also affects mitochondrial phospholipid dynamics and overall mitochondrial function that influences skeletal muscle activity (8, 67). PI3K/Akt signaling plays a key role as a general regulator of skeletal muscle homeostasis (including protein synthesis and degradation) in skeletal muscle tissues. Multiple factors (e.g., TNF-*α* and IL-6) can promote skeletal muscle depletion by inhibiting the PI3K/Akt signaling pathway in cancer cachexia. In addition, oxidative stress promotes skeletal muscle depletion by increasing protein degradation and inducing myofiber apoptosis through damage to mitochondrial DNA (68, 69). Therefore, decreasing the secretion of associated factors and reducing inflammation are possible therapeutic ideas.

From a therapeutic aspect, sarcopenia is a strong adverse prognostic factor in patients with a variety of cancers including uroepithelial carcinoma (68). Chemotherapy regimens for BCa may further accelerate muscle wasting and lead to weight loss, e.g., cisplatin may cause muscle dysfunction by altering a variety of mechanisms including the ubiquitin-protein pathway, calcium homeostasis, mitochondrial damage, and cytokine upregulation (70). For patients treated with neoadjuvant chemotherapy, Tobias Tuse Dunk Hansen et al. observed a higher prevalence of skeletal sarcopenia in patients receiving NAC compared with patients undergoing surgery alone or in combination with NAC (71). Timothy D. Lyon et al. demonstrated that patients with sarcopenia who received neoadjuvant chemotherapy prior to radical cystectomy were associated with poorer CSS, suggesting that that sarcopenia is associated with NAC prognosis, but this does not suggest that skeletal muscle reduction is significantly associated with pathologic response to chemotherapy. Pierre Regnier et al. showed that skeletal muscle reduction was an independent predictor associated with risk of renal damage during NAC and early postoperative complications after RC (72).

Triple therapy (TMT) is a well-established alternative to radical cystectomy (RC) for patients with muscle-invasive bladder cancer (MIBC) seeking to preserve their native bladder or who are inoperable due to comorbidities. Fukushima et al. demonstrated through a literature response that sarcopenia does not affect the response and prognosis of triple therapy in patients with MIBC on bladder-preserving therapy. However, the effect of sarcopenia on the complication rate of bladder preserving therapy is uncertain due to limited evidence (73, 125). Liu et al. showed that both sarcopenia and a high systemic immunoinflammatory index (SII) were useful predictors of response to intravesical BCG in intermediate- and high-risk NMIBC patients (74). Intermediate- and high-risk NMIBC patients with sarcopenia or high SII at diagnosis are associated with poorer RFS, and the combination of sarcopenia and SII may be a better predictor of RFS. Ferini et al. demonstrated that sarcopenia could not be considered a negative prognostic factor for elderly patients with MIBC receiving radiotherapy (75). Stangl-Kremser et al. also concluded that that sarcopenia has no prognostic effect on survival in patients with high-risk urothelial carcinoma of the bladder undergoing radiotherapy (76). Thus, radiotherapy is a viable and effective option for these patients, especially if surgery is not indicated.

Not only that, but there is a potential impact of sarcopenia on the surgical management of bladder cancer patients. Several studies have reported the prognostic role of skeletal muscle reduction in patients undergoing radical cystectomy for bladder cancer. Studies have shown that skeletal muscle reduction is a significant predictor of cancer-specific survival (CSS) and overall survival (OS). Psutka et al. demonstrated, for the first time, that skeletal muscle reduction was an independent predictor of poor CSS and OS in bladder cancer. Patients with skeletal muscle reduction had lower 5-year CSS and OS rates than non-skeletal muscle reduction patients (49% vs. 72% for CSS and 39% vs. 70% for OS) (77). Similar results were reported in the studies by Shimpei Yamashita and Roman Mayr et al. (78, 79). In conclusion, it has been shown through most studies that skeletal sarcopenia is an important factor in the poor prognosis of patients undergoing radical cystectomy for bladder cancer (80, 81).

### 4.2 Prostate cancer

Prostate cancer is one of the most common malignant tumors of the male reproductive system, the second most common cancer in men and the fifth leading cause of cancer death (82). The prognostic value of sarcopenia in advanced prostate cancer has been evaluated, and the study showed that sarcopenia was significantly associated with progression-free survival in advanced prostate cancer (HR = 1.61, 95% CI: 1.26, 2.06, *p* < 0.01), but sarcopenia did not have a significant effect on overall survival and cancer-specific survival, suggesting that sarcopenia is a an important prognostic factor for progression-free survival in patients with advanced PCa (81).

Androgen deprivation therapy (ADT) stands as the current treatment modality for metastatic prostate cancer; however, the majority of cases ultimately advance toward desmoplasia-resistant prostate cancer typified by the escalation of prostate-specific antigen levels and the progression of both primary and metastatic sites. Given the chronic nature of prostate cancer, these therapies tend to be administered over an extended duration, thereby establishing a strong association between long-term chemotherapy and the development of sarcopenia. Consequently, the urgent need to investigate the relationship between chemotherapy for prostate cancer and sarcopenia emerges as a paramount clinical concern that significantly impacts the prognosis of patients undergoing chemotherapy for prostate cancer. ADT therapies significantly impact the human body, inducing side effects such as skeletal sarcopenia and bone loss, notably among the elderly demographic (83). Active intervention can successfully mitigate these adverse effects posed by ADT therapy. On a mechanistic level, sarcopenia accelerated by hormone deprivation therapy is a result of prostate tumor-derived growth differentiation factor 11 (GDF11) signaling from the tumor to the muscle tissue (84). Liver-targeted testosterone therapy (LTTT) presents a promising, simplistic approach to prevent sarcopenia and bone loss during ADT (85). Furthermore, Zhang and his team proposed the potential of eldecalcitol to counteract sarcopenia caused by ADT treatment, utilizing the PI3K/AKT/FOXOs signaling pathway in a constructed mouse model (86). Also, a resistance-focused exercise regimen proves effective in ameliorating sarcopenia in men with prostate cancer undergoing ADT (87, 88).

The impact of sarcopenia, a negative prognostic factor, on prostate cancer treated with docetaxel or abiraterone acetate has been confirmed through a series of clinical trials (89–92). Additional research implies that sarcopenia may interact with excessive visceral fat accumulation, thereby adversely affecting early urinary function following I-125 low-dose brachytherapy against prostate cancer (93). However, it is important to note that such acceleration in sarcopenia was not observed in men with metastatic castration-resistant prostate cancer (mCRPC) undergoing Ra-223 treatment (94). Furthermore, a related study that explored the correlation between sarcopenia in castration-resistant prostate cancer (CRPC) and treatment outcomes with androgen receptor axial therapy (ARATs) revealed that the latter could potentially offer enhanced efficacy among CRPC patients with sarcopenia, compared to those devoid of it (95).

Furthermore, the prognosis of prostate cancer patients who are undergoing surgical intervention varies significantly between those with sarcopenia and those sans sarcopenia. According to research conducted by Mitsui Y et al., patients diagnosed with sarcopenia have reportedly expressed greater clinical dissatisfaction concerning postoperative urinary function than their counterparts lacking this condition, in the context of robot-assisted radical prostatectomy (96). It is plausible that sarcopenia might serve as a predictive factor for postoperative erectile dysfunction following robot-assisted radical prostatectomy (97). Mason et al. showed that sarcopenia did not predict biochemical recurrence in patients undergoing radical prostatectomy (98). In contrast, Pak et al. showed that preoperative sarcopenia led to a higher risk of biochemical recurrence in patients undergoing radical prostatectomy (99). More clinical samples are needed to demonstrate the link between sarcopenia and biochemical recurrence of prostate cancer.

### 4.3 Renal cell cancer

Globally, Renal Cell Carcinoma (RCC) is ranked as the sixth and tenth most prevalent forms of cancer in males and females, respectively (100). A meta-analysis highlighted a significant correlation between reductions in skeletal muscle (sarcopenia) and Overall Survival (OS), Cancer-Specific Survival (CSS), and Progression-Free Survival (PFS) of RCC patients regardless of variables like age, tumor location, and stage (101). Notably, the study also suggested a persistent and potentially intensifying implication of skeletal muscle reduction on all-cause mortality and cancer-specific mortality throughout prolonged follow-ups in RCC. Nonetheless, this inference may be skewed, as the researchers could not unequivocally attribute the survival outcomes to tumor development or skeletal muscle reduction, a challenge that has baffled previous studies as well. Another meta-analysis reiterated these inconclusive results, showing no significant difference in PFS between sarcopenic and non-sarcopenic RCC patients. Consequently, supplemental data-oriented studies are necessitated (102).

Sarcopenia is a significant prognostic factor in metastatic renal cell carcinoma (103). In patients diagnosed with metastatic renal cell carcinoma undergoing chemotherapy, the presence of skeletal sarcopenia could potentially heighten the risk of treatment-related toxicity while simultaneously shortening survival rates. This finding originates from a retrospective analysis conducted by Hideto Ueki et al., utilizing the Skeletal Muscle Minority Index (SMI) as a predictor for the therapeutic efficacy of nivolumab in the treatment of this disease. Concurrently, the study draws parallels between sarcopenia - diagnosed via the Psoas Muscle Index (PMI) - and an unfavorable prognosis in patients diagnosed with RCC. It is important to underscore, however, the considerable variation in diagnosis rates of sarcopenia and the lack of a demonstrable association between sarcopenia, as identified by SMI, and prognosis (104, 105). During the course of cabozantinib therapy, it has been observed that a considerable proportion of patients manifested significant early skeletal muscle deterioration correlating to an unfavorable progression-free survival (PFS) (106, 107). Furthermore, myasthenia gravis emerged as a crucial prognostic indicator in metastatic renal cell carcinoma (mRCC) patients undergoing primary treatment with sunitinib (108). Additionally, a study by S. Antoun et al., demonstrated that patients displaying sarcopenia were prone to encounter dose-limiting adverse events during targeted therapy regimes, as indicated by clinical data presenting skeletal muscle loss as a predictor of such therapies’ toxicity. Notably, a body mass index (BMI) less than 25 kg/m^2 coupled with diminished muscle mass emerged as a significant predictor of targeted agents’ toxicity (109–111). Intriguingly, sarcopenia was found to predict the response to Interleukin-2 (IL-2) treatment in metastatic RCC scenarios (112).

Jongpil Lee and collaborators explored the correlation between the decrement in skeletal muscle mass and the overall survival rate in patients undergoing radical surgery for localized renal cell carcinoma (113). The study implicates that sarcopenia in conjunction with a modified Glasgow scale could serve as a more robust prognostic marker following the surgical procedures for localized renal cell carcinoma (114). Additionally, limited mobility, when coupled with serum albumin levels, could also prefigure the prognosis after surgery (115, 116). A separate study by Pranav Sharma et al. postulated that hypokinesia might act as a potential prognostic indicator for overall survival subsequent to a nephrectomy for metastatic renal cell carcinoma (117, 118). Moreover, a study conducted on a Chinese cohort further substantiated that sarcopenia post nephrectomy is indicative of a poor prognosis (119). Additionally, a significant correlation has been found between sarcopenia and an increased risk of recurrence of clear cell renal cell carcinoma in male patients (120). Consequently, emergent prognostic tools, including the novel index derived from the integration of albumin-globulin score and sarcopenia, known as the CAS, have been developed to forecast the progression of renal cancer subsequent to a surgical intervention (121). Furthermore, the low ratio of creatinine to cystatin-C (Cr/Cys-C) could potentially function as a serum biomarker indicating the development of sarcopenia in patients undergoing nephrectomy treatment for RCC (122).

### 4.4 Germ cell carcinoma of the testis (GCT)

GCT is a common solid tumor among young men that is sensitive to chemotherapy and has a high cure rate. Patients with GCT are characterized by their general youth and are therefore unlikely to have aging-related muscle loss at the time of diagnosis. Phuong et al. showed that in patients with testicular germ cell carcinoma (GCT) receiving cytotoxic chemotherapy, decreased skeletal muscle mass during chemotherapy was independently associated with a higher incidence of chemotherapy-related adverse events (123). Therefore, intervening in GCT patients with decreased skeletal muscle mass during chemotherapy may be able to reduce the incidence of adverse events. In addition, a prospective study found that reduced muscle mass was significantly associated with poor postoperative prognosis in patients with metastatic germ cell tumors (mGCTs) receiving postoperative chemotherapy who underwent post-chemotherapy retroperitoneal lymphadenectomy (PC-RPLND) after oncolytic chemotherapy, and that further evaluation of the preoperative nutritional status of this population may be helpful in reducing morbidity after PC-RPLND (124).

## 5 Conclusion

In recent years, there has been an increasing amount of research on skeletal sarcopenia and urologic diseases, and the importance of sarcopenia in urologic diseases has received increasing attention. Sarcopenia and urologic diseases interact with each other; urologic diseases cause sarcopenia, and sarcopenia aggravates the condition of the original disease, thus falling into a vicious circle. This review systematically analyzes the relationship between sarcopenia and urological diseases and reveals the potential role of sarcopenia in the development and progression of urological diseases. Our analysis suggests that sarcopenia is not only a common complication of urologic diseases, but may also be an important predictor of disease progression and poor prognosis. And this review highlights potential interventions for sarcopenia, such as nutritional support and physical activity, which may help improve the prognosis of patients with urologic diseases. Although our review provides valuable insights, we also recognize that limitations exist. First, the included studies were diverse in design, which may have contributed to the heterogeneity of results. Second, most of the studies were cross-sectional, and future studies need to be further validated using more uniform study designs and methods, as well as prospective studies with large samples. Early screening, diagnosis, and intervention of sarcopenia in urologic diseases is important. Therefore, we suggest that recommendations for screening and management of sarcopenia be included in clinical guidelines for urologic diseases. In conclusion, this review provides new perspectives for understanding the role of sarcopenia in urologic diseases and provides valuable information for future research and clinical practice.

## References

[1] Yuan S Larsson SC. Epidemiology of sarcopenia: prevalence, risk factors, and consequences. Metabolism. (2023) 144:155533. doi: 10.1016/j.metabol.2023.155533, PMID: 36907247

[2] Cruz-Jentoft AJ Sayer AA. Sarcopenia [published correction appears in lancet. 2019 Jun 29;393(10191):2590]. Lancet. (2019) 393:2636–46. doi: 10.1016/S0140-6736(19)31138-9 31171417

[3] Livshits G Kalinkovich A. A cross-talk between sestrins, chronic inflammation and cellular senescence governs the development of age-associated sarcopenia and obesity. Ageing Res Rev. (2023) 86:101852. doi: 10.1016/j.arr.2023.101852, PMID: 36642190

[4] Pan L Xie W Fu X Lu W Jin H Lai J. Inflammation and sarcopenia: a focus on circulating inflammatory cytokines. Exp Gerontol. (2021) 154:111544. doi: 10.1016/j.exger.2021.111544, PMID: 34478826

[5] IdaSKanekoRNagataHNoguchiYArakiYNakaiM. Association between sarcopenia and overactive bladder in elderly diabetic patients. J Nutr Health Aging. (2019) 23:532–7. doi: 10.1007/s12603-019-1190-1, PMID: 31233074

[6] Stenvinkel P Larsson TE. Chronic kidney disease: a clinical model of premature aging. Am J Kidney Dis. (2013) 62:339–51. doi: 10.1053/j.ajkd.2012.11.051 23357108

[7] Kovač MB Pavlin T Čavka L Ribnikar D Spazzapan S Templeton AJ. The trajectory of sarcopenia following diagnosis of prostate cancer: a systematic review and meta-analysis. J Geriatr Oncol. (2023) 14:101594. doi: 10.1016/j.jgo.2023.101594, PMID: 37482497

[8] Antunes D Padrão AI Maciel E Santinha D Oliveira P Vitorino R. Molecular insights into mitochondrial dysfunction in cancer-related muscle wasting. Biochim Biophys Acta. (2014) 1841:896–905. doi: 10.1016/j.bbalip.2014.03.004, PMID: 24657703

[9] Wang K Liu Q Tang M Qi G Qiu C Huang Y. Chronic kidney disease-induced muscle atrophy: molecular mechanisms and promising therapies. Biochem Pharmacol. (2023) 208:115407. doi: 10.1016/j.bcp.2022.115407, PMID: 36596414

[10] Rosenberg IH. Sarcopenia: origins and clinical relevance. J Nutr. (1997) 127:990S–1S. doi: 10.1093/jn/127.5.990S, PMID: 9164280

[11] Cruz-Jentoft AJ Baeyens JP Bauer JM Boirie Y Cederholm T Landi F. Sarcopenia: European consensus on definition and diagnosis: report of the European working group on sarcopenia in older people. Age Ageing. (2010) 39:412–23. doi: 10.1093/ageing/afq034, PMID: 20392703 PMC2886201

[12] Cruz-Jentoft AJ Bahat G Bauer J Boirie Y Bruyère O Cederholm T. Sarcopenia: revised European consensus on definition and diagnosis [published correction appears in age ageing. 2019 Jul 1;48(4):601]. Age Ageing. (2019) 48:16–31. doi: 10.1093/ageing/afy169, PMID: 31081853 PMC6593317

[13] Muscaritoli M Anker SD Argilés J Aversa Z Bauer JM Biolo G. Consensus definition of sarcopenia, cachexia and pre-cachexia: joint document elaborated by special interest groups (SIG) "cachexia-anorexia in chronic wasting diseases" and "nutrition in geriatrics". Clin Nutr. (2010) 29:154–9. doi: 10.1016/j.clnu.2009.12.004, PMID: 20060626

[14] Fielding RA Vellas B Evans WJ Bhasin S Morley JE Newman AB. Sarcopenia: an undiagnosed condition in older adults. Current consensus definition: prevalence, etiology, and consequences. International working group on sarcopenia. J Am Med Dir Assoc. (2011) 12:249–56. doi: 10.1016/j.jamda.2011.01.003, PMID: 21527165 PMC3377163

[15] Morley JE Abbatecola AM Argiles JM Baracos V Bauer J Bhasin S. Sarcopenia with limited mobility: an international consensus. J Am Med Dir Assoc. (2011) 12:403–9. doi: 10.1016/j.jamda.2011.04.014, PMID: 21640657 PMC5100674

[16] Studenski SA Peters KW Alley DE Cawthon PM McLean RR Harris TB. The FNIH sarcopenia project: rationale, study description, conference recommendations, and final estimates. J Gerontol A Biol Sci Med Sci. (2014) 69:547–58. doi: 10.1093/gerona/glu010, PMID: 24737557 PMC3991146

[17] Buckinx F Landi F Cesari M Fielding RA Visser M Engelke K. Pitfalls in the measurement of muscle mass: a need for a reference standard. J Cachexia Sarcopenia Muscle. (2018) 9:269–78. doi: 10.1002/jcsm.12268, PMID: 29349935 PMC5879987

[18] MasanésFLuqueRILuqueXRISalvàASerra-RexachJAArtazaI. Cut-off points for muscle mass – not grip strength or gait speed - determine variations in sarcopenia prevalence. J Nutr Health Aging. (2017) 21:825–9. doi: 10.1007/s12603-016-0844-528717813

[19] Treviño-Aguirre E López-Teros T Gutiérrez-Robledo L Vandewoude M Pérez-Zepeda M. Availability and use of dual energy X-ray absorptiometry (DXA) and bio-impedance analysis (BIA) for the evaluation of sarcopenia by Belgian and Latin American geriatricians. J Cachexia Sarcopenia Muscle. (2014) 5:79–81. doi: 10.1007/s13539-013-0126-6, PMID: 24442632 PMC3953316

[20] Chen LK Liu LK Woo J Assantachai P Auyeung TW Bahyah KS. Sarcopenia in Asia: consensus report of the Asian working Group for Sarcopenia. J Am Med Dir Assoc. (2014) 15:95–101. doi: 10.1016/j.jamda.2013.11.025, PMID: 24461239

[21] Chen LK Woo J Assantachai P Auyeung TW Chou MY Iijima K. Asian working Group for Sarcopenia: 2019 consensus update on sarcopenia diagnosis and treatment. J Am Med Dir Assoc. (2020) 21:300–307.e2. doi: 10.1016/j.jamda.2019.12.012, PMID: 32033882

[22] Wang M Tan Y Shi Y Wang X Liao Z Wei P. Diabetes and Sarcopenic obesity: pathogenesis, diagnosis, and treatments. Front Endocrinol. (2020) 11:568. doi: 10.3389/fendo.2020.00568, PMID: 32982969 PMC7477770

[23] Donini LM Busetto L Bischoff SC Cederholm T Ballesteros-Pomar MD Batsis JA. Definition and diagnostic criteria for Sarcopenic obesity: ESPEN and EASO consensus statement. Obes Facts. (2022) 15:321–35. doi: 10.1159/000521241, PMID: 35196654 PMC9210010

[24] Maggio M Ceda GP Ticinesi A de Vita F Gelmini G Costantino C. Instrumental and non-instrumental evaluation of 4-meter walking speed in older individuals. PLoS One. (2016) 11:e0153583. doi: 10.1371/journal.pone.0153583, PMID: 27077744 PMC4831727

[25] Welch SA Ward RE Beauchamp MK Leveille SG Travison T Bean JF. The short physical performance battery (SPPB): a quick and useful tool for fall risk stratification among older primary care patients. J Am Med Dir Assoc. (2021) 22:1646–51. doi: 10.1016/j.jamda.2020.09.038, PMID: 33191134 PMC8113335

[26] Podsiadlo D Richardson S. The timed "up & go": a test of basic functional mobility for frail elderly persons. J Am Geriatr Soc. (1991) 39:142–8. doi: 10.1111/j.1532-5415.1991.tb01616.x 1991946

[27] Ibrahim K May C Patel HP Baxter M Sayer AA Roberts H. A feasibility study of implementing grip strength measurement into routine hospital practice (GRImP): study protocol. Pilot Feasibility Stud. (2016) 2:27. doi: 10.1186/s40814-016-0067-x, PMID: 27965846 PMC5154137

[28] Beaudart C McCloskey E Bruyère O Cesari M Rolland Y Rizzoli R. Sarcopenia in daily practice: assessment and management. BMC Geriatr. (2016) 16:170. doi: 10.1186/s12877-016-0349-4, PMID: 27716195 PMC5052976

[29] Kim EY Kim YS Park I Ahn HK Cho EK Jeong YM. Prognostic significance of CT-determined sarcopenia in patients with small-cell lung Cancer. J Thorac Oncol. (2015) 10:1795–9. doi: 10.1097/JTO.0000000000000690, PMID: 26484630

[30] Hull H He Q Thornton J Javed F Allen L Wang J. iDXA, prodigy, and DPXL dual-energy X-ray absorptiometry whole-body scans: a cross-calibration study. J Clin Densitom. (2009) 12:95–102. doi: 10.1016/j.jocd.2008.09.004, PMID: 19028125 PMC2661815

[31] Kyle UG Genton L Hans D Pichard C. Validation of a bioelectrical impedance analysis equation to predict appendicular skeletal muscle mass (ASMM). Clin Nutr. (2003) 22:537–43. doi: 10.1016/s0261-5614(03)00048-7, PMID: 14613755

[32] Perkisas S Bastijns S Sanchez-Rodriguez D Piotrowicz K De Cock AM full SARCUS working group. Application of ultrasound for muscle assessment in sarcopenia: 2020 SARCUS update: reply to the letter to the editor: SARCUS working group on behalf of the sarcopenia special interest Group of the European Geriatric Medicine Society. Eur Geriatr Med. (2021) 12:427–8. doi: 10.1007/s41999-021-00462-y 33595779

[33] Nagae M Umegaki H Yoshiko A Fujita K Komiya H Watanabe K. Echo intensity is more useful in predicting hospital-associated complications than conventional sarcopenia-related parameters in acute hospitalized older patients. Exp Gerontol. (2021) 150:111397. doi: 10.1016/j.exger.2021.111397, PMID: 33965558

[34] Baumgartner RN Koehler KM Gallagher D Romero L Heymsfield SB Ross RR. Epidemiology of sarcopenia among the elderly in New Mexico [published correction appears in am J Epidemiol 1999;149(12):1161]. Am J Epidemiol. (1998) 147:755–63. doi: 10.1093/oxfordjournals.aje.a009520, PMID: 9554417

[35] Yuan Q Hu J Yuan F An J. Predictive role of pretreatment skeletal muscle mass index for long-term survival of bladder cancer patients: a meta-analysis. PLoS One. (2023) 18:e0288077. doi: 10.1371/journal.pone.0288077, PMID: 37390088 PMC10313011

[36] Albersheim J Sathianathen NJ Zabell J Renier J Bailey T Hanna P. Skeletal muscle and fat mass indexes predict discharge disposition after radical cystectomy. J Urol. (2019) 202:1143–9. doi: 10.1097/JU.0000000000000450, PMID: 31483713

[37] Hsu WH Ko AT Weng CS Chang CL Jan YT Lin JB. Explainable machine learning model for predicting skeletal muscle loss during surgery and adjuvant chemotherapy in ovarian cancer. J Cachexia Sarcopenia Muscle. (2023) 14:2044–53. doi: 10.1002/jcsm.13282, PMID: 37435785 PMC10570082

[38] Somasundaram E Castiglione JA Brady SL Trout AT. Defining Normal ranges of skeletal muscle area and skeletal muscle index in children on CT using an automated deep learning pipeline: implications for sarcopenia diagnosis. AJR Am J Roentgenol. (2022) 219:326–36. doi: 10.2214/AJR.21.27239 35234481

[39] McCarthy C Schoeller D Brown JC Gonzalez MC Varanoske AN Cataldi D. D3-creatine dilution for skeletal muscle mass measurement: historical development and current status. J Cachexia Sarcopenia Muscle. (2022) 13:2595–607. doi: 10.1002/jcsm.13083, PMID: 36059250 PMC9745476

[40] Shankaran M Czerwieniec G Fessler C Wong PYA Killion S Turner SM. Dilution of oral D3-Creatine to measure creatine pool size and estimate skeletal muscle mass: development of a correction algorithm. J Cachexia Sarcopenia Muscle. (2018) 9:540–6. doi: 10.1002/jcsm.12278, PMID: 29663711 PMC5989770

[41] Clark RV Walker AC Miller RR O'Connor-Semmes RL Ravussin E Cefalu WT. Creatine (methyl-d_3_) dilution in urine for estimation of total body skeletal muscle mass: accuracy and variability vs. MRI and DXA. J Appl Physiol (1985). (2018) 124:1–9. doi: 10.1152/japplphysiol.00455.2016, PMID: 28860169 PMC6048459

[42] Buehring B Siglinsky E Krueger D Evans W Hellerstein M Yamada Y. Comparison of muscle/lean mass measurement methods: correlation with functional and biochemical testing. Osteoporos Int. (2018) 29:675–83. doi: 10.1007/s00198-017-4315-6, PMID: 29198074

[43] Curcio F Ferro G Basile C Liguori I Parrella P Pirozzi F. Biomarkers in sarcopenia: a multifactorial approach. Exp Gerontol. (2016) 85:1–8. doi: 10.1016/j.exger.2016.09.007, PMID: 27633530

[44] Aluganti Narasimhulu C Singla DK. Amelioration of diabetes-induced inflammation mediated pyroptosis, sarcopenia, and adverse muscle remodelling by bone morphogenetic protein-7. J Cachexia Sarcopenia Muscle. (2021) 12:403–20. doi: 10.1002/jcsm.12662, PMID: 33463042 PMC8061343

[45] Ma W Zhang R Huang Z Zhang Q Xie X Yang X. PQQ ameliorates skeletal muscle atrophy, mitophagy and fiber type transition induced by denervation via inhibition of the inflammatory signaling pathways. Ann Transl Med. (2019) 7:440. doi: 10.21037/atm.2019.08.101, PMID: 31700876 PMC6803183

[46] Sun H Sun J Li M Qian L Zhang L Huang Z. Transcriptome analysis of immune receptor activation and energy metabolism reduction as the underlying mechanisms in Interleukin-6-induced skeletal muscle atrophy. Front Immunol. (2021) 12:730070. doi: 10.3389/fimmu.2021.730070, PMID: 34552592 PMC8450567

[47] Fang WY Tseng YT Lee TY Fu YC Chang WH Lo WW. Triptolide prevents LPS-induced skeletal muscle atrophy via inhibiting NF-κB/TNF-α and regulating protein synthesis/degradation pathway. Br J Pharmacol. (2021) 178:2998–3016. doi: 10.1111/bph.15472, PMID: 33788266

[48] Bano G Trevisan C Carraro S Solmi M Luchini C Stubbs B. Inflammation and sarcopenia: a systematic review and meta-analysis. Maturitas. (2017) 96:10–5. doi: 10.1016/j.maturitas.2016.11.006 28041587

[49] Huang Z Zhong L Zhu J Xu H Ma W Zhang L. Inhibition of IL-6/JAK/STAT3 pathway rescues denervation-induced skeletal muscle atrophy [published correction appears in Ann Transl med]. Ann Transl Med. (2020) 8:1681. doi: 10.21037/atm-20-7269, PMID: 33490193 PMC7812230

[50] Shen Y Li M Wang K Qi G Liu H Wang W. Diabetic muscular atrophy: molecular mechanisms and promising therapies. Front Endocrinol. (2022) 13:917113. doi: 10.3389/fendo.2022.917113, PMID: 35846289 PMC9279556

[51] Huang Y Wang B Hassounah F Price SR Klein J Mohamed TMA. The impact of senescence on muscle wasting in chronic kidney disease. J Cachexia Sarcopenia Muscle. (2023) 14:126–41. doi: 10.1002/jcsm.13112, PMID: 36351875 PMC9891952

[52] DeAndrade J Pedersen M Garcia L Nau P. Sarcopenia is a risk factor for complications and an independent predictor of hospital length of stay in trauma patients. J Surg Res. (2018) 221:161–6. doi: 10.1016/j.jss.2017.08.018, PMID: 29229123

[53] SongWHuHNiJZhangHZhangYZhangH. The role of sarcopenia in overactive bladder in adults in the United States: retrospective analysis of NHANES 2011-2018. J Nutr Health Aging. (2023) 27:734–40. doi: 10.1007/s12603-023-1972-3, PMID: 37754213

[54] Hashimoto M Shimizu N Nishimoto M Minami T Fujita K Yoshimura K. Sarcopenia and visceral obesity are significantly related to severe storage symptoms in geriatric female patients. Res Rep Urol. (2021) 13:557–63. doi: 10.2147/RRU.S321323, PMID: 34395328 PMC8357624

[55] Wu CH Lai TS Chen YM Chen CM Yang SC Liang PC. Quantification of abdominal muscle mass and diagnosis of sarcopenia with cross-sectional imaging in patients with polycystic kidney disease: correlation with Total kidney volume. Diagnostics. (2022) 12:755. doi: 10.3390/diagnostics12030755, PMID: 35328308 PMC8947181

[56] Lee J Ryu H Kim YC Park HC Ahn C Lee KB. Nutritional status is associated with preserved kidney function in patients with autosomal dominant polycystic kidney disease. J Ren Nutr. (2023) 33:529–37. doi: 10.1053/j.jrn.2023.02.006, PMID: 36965751

[57] Mannaa M Pfennigwerth P Fielitz J Gollasch M Boschmann M. Mammalian target of rapamycin inhibition impacts energy homeostasis and induces sex-specific body weight loss in humans. J Cachexia Sarcopenia Muscle. (2023) 14:2757–67. doi: 10.1002/jcsm.13352, PMID: 37897143 PMC10751400

[58] Ryu H Yang YJ Kang E Ahn C Yang SJ Oh KH. Greater adherence to the dietary approaches to stop hypertension dietary pattern is associated with preserved muscle strength in patients with autosomal dominant polycystic kidney disease: a single-center cross-sectional study. Nutr Res. (2021) 93:99–110. doi: 10.1016/j.nutres.2021.07.006, PMID: 34461351

[59] Zhang Y Tian C Wang Y Zhang H Ni J Song W. Association between sarcopenia and kidney stones in United States adult population between 2011 and 2018. Front Nutr. (2023) 10:1123588. doi: 10.3389/fnut.2023.1123588, PMID: 36950333 PMC10025351

[60] Song W Hu H Ni J Zhang H Zhang H Lu J. Prognostic value of total body muscle-fat ratio in patients with kidney stone disease: a US population-based study. Heliyon. (2023) 9:e20339. doi: 10.1016/j.heliyon.2023.e20339, PMID: 37810105 PMC10560043

[61] Lobo N Afferi L Moschini M Mostafid H Porten S Psutka SP. Epidemiology, screening, and prevention of bladder Cancer. Eur Urol Oncol. (2022) 5:628–39. doi: 10.1016/j.euo.2022.10.003 36333236

[62] Zhu S Yu W Yang X Wu C Cheng F. Traditional classification and novel subtyping systems for Bladder Cancer. Front Oncol. (2020) 10:102. doi: 10.3389/fonc.2020.00102, PMID: 32117752 PMC7025453

[63] Chang SS Boorjian SA Chou R Clark PE Daneshmand S Konety BR. Diagnosis and treatment of non-muscle invasive bladder Cancer: AUA/SUO guideline. J Urol. (2016) 196:1021–9. doi: 10.1016/j.juro.2016.06.049, PMID: 27317986

[64] Patel VG Oh WK Galsky MD. Treatment of muscle-invasive and advanced bladder cancer in 2020. CA Cancer J Clin. (2020) 70:404–23. doi: 10.3322/caac.21631 32767764

[65] Sui X Lei L Chen L Xie T Li X. Inflammatory microenvironment in the initiation and progression of bladder cancer. Oncotarget. (2017) 8:93279–94. doi: 10.18632/oncotarget.21565, PMID: 29190997 PMC5696263

[66] Shadpour P Zamani M Aghaalikhani N Rashtchizadeh N. Inflammatory cytokines in bladder cancer. J Cell Physiol. (2019) 234:14489–99. doi: 10.1002/jcp.28252 30779110

[67] Padrão AI Oliveira P Vitorino R Colaço B Pires MJ Márquez M. Bladder cancer-induced skeletal muscle wasting: disclosing the role of mitochondria plasticity. Int J Biochem Cell Biol. (2013) 45:1399–409. doi: 10.1016/j.biocel.2013.04.014, PMID: 23608519

[68] Fukushima H Fujii Y Koga F. Metabolic and molecular basis of sarcopenia: implications in the Management of Urothelial Carcinoma. Int J Mol Sci. (2019) 20:760. doi: 10.3390/ijms20030760, PMID: 30754663 PMC6387186

[69] Yeom E Yu K. Understanding the molecular basis of anorexia and tissue wasting in cancer cachexia. Exp Mol Med. (2022) 54:426–32. doi: 10.1038/s12276-022-00752-w, PMID: 35388147 PMC9076846

[70] Conte E Bresciani E Rizzi L Cappellari O de Luca A Torsello A. Cisplatin-induced skeletal muscle dysfunction: mechanisms and counteracting therapeutic strategies. Int J Mol Sci. (2020) 21:1242. doi: 10.3390/ijms21041242, PMID: 32069876 PMC7072891

[71] Hansen TTD Omland LH von Heymann A Johansen C Clausen MB Suetta C. Development of sarcopenia in patients with bladder Cancer: a systematic review. Semin Oncol Nurs. (2021) 37:151108. doi: 10.1016/j.soncn.2020.151108, PMID: 33431235

[72] Lyon TD Frank I Takahashi N Boorjian SA Moynagh MR Shah PH. Sarcopenia and response to neoadjuvant chemotherapy for muscle-invasive bladder Cancer. Clin Genitourin Cancer. (2019) 17:216–222.e5. doi: 10.1016/j.clgc.2019.03.007, PMID: 31060857

[73] Fukushima H Koga F. Impact of sarcopenia in bladder preservation therapy for muscle-invasive bladder cancer patients: a narrative review. Transl Androl Urol. (2022) 11:1433–41. doi: 10.21037/tau-22-355, PMID: 36386266 PMC9641057

[74] Liu P Chen S Gao X Liang H Sun D Shi B. Preoperative sarcopenia and systemic immune-inflammation index can predict response to intravesical Bacillus Calmette-Guerin instillation in patients with non-muscle invasive bladder cancer. Front Immunol. (2022) 13:1032907. doi: 10.3389/fimmu.2022.1032907, PMID: 36225922 PMC9549861

[75] Ferini G Cacciola A Parisi S Lillo S Molino L Tamburella C. Curative radiotherapy in elderly patients with muscle invasive bladder Cancer: the prognostic role of sarcopenia. In Vivo. (2021) 35:571–8. doi: 10.21873/invivo.12293, PMID: 33402511 PMC7880729

[76] Stangl-Kremser J D'Andrea D Vartolomei M Abufaraj M Goldner G Baltzer P. Prognostic value of nutritional indices and body composition parameters including sarcopenia in patients treated with radiotherapy for urothelial carcinoma of the bladder. Urol Oncol. (2019) 37:372–9. doi: 10.1016/j.urolonc.2018.11.001, PMID: 30578161

[77] Psutka SP Carrasco A Schmit GD Moynagh MR Boorjian SA Frank I. Sarcopenia in patients with bladder cancer undergoing radical cystectomy: impact on cancer-specific and all-cause mortality. Cancer. (2014) 120:2910–8. doi: 10.1002/cncr.28798, PMID: 24840856

[78] Yamashita S Iguchi T Koike H Wakamiya T Kikkawa K Kohjimoto Y. Impact of preoperative sarcopenia and myosteatosis on prognosis after radical cystectomy in patients with bladder cancer. Int J Urol. (2021) 28:757–62. doi: 10.1111/iju.14569, PMID: 33821510

[79] Mayr R Gierth M Zeman F Reiffen M Seeger P Wezel F. Sarcopenia as a comorbidity-independent predictor of survival following radical cystectomy for bladder cancer. J Cachexia Sarcopenia Muscle. (2018) 9:505–13. doi: 10.1002/jcsm.12279, PMID: 29479839 PMC5989852

[80] Ibilibor C Psutka SP Herrera J Rivero JR Wang H Farrell AM. The association between sarcopenia and bladder cancer-specific mortality and all-cause mortality after radical cystectomy: a systematic review and meta-analysis. Arab J Urol. (2021) 19:98–103. doi: 10.1080/2090598X.2021.1876289, PMID: 33763255 PMC7954502

[81] de Pablos-Rodríguez P Del Pino-Sedeño T Infante-Ventura D de Armas-Castellano A Backhaus MR Ferrer JFL. Prognostic impact of sarcopenia in patients with advanced prostate carcinoma: a systematic review. J Clin Med. (2022) 12:57. doi: 10.3390/jcm12010057, PMID: 36614862 PMC9821501

[82] Bray F Laversanne M Sung H Ferlay J Siegel RL Soerjomataram I. Global cancer statistics 2022: GLOBOCAN estimates of incidence and mortality worldwide for 36 cancers in 185 countries. CA Cancer J Clin. (2024) 74:229–63. doi: 10.3322/caac.21834, PMID: 38572751

[83] Bargiota A Oeconomou A Zachos I Samarinas M Pisters LL Tzortzis V. Adverse effects of androgen deprivation therapy in patients with prostate cancer: focus on muscle and bone health. J BUON. (2020) 25:1286–94. PMID: 32862568

[84] Pan C Jaiswal Agrawal N Zulia Y Singh S Sha K Mohler JL. Prostate tumor-derived GDF11 accelerates androgen deprivation therapy-induced sarcopenia. JCI Insight. (2020) 5:e127018. doi: 10.1172/jci.insight.127018, PMID: 32078585 PMC7213789

[85] Rhee H Navaratnam A Oleinikova I Gilroy D Scuderi Y Heathcote P. A novel liver-targeted testosterone therapy for sarcopenia in androgen deprived men with prostate Cancer. J Endocr Soc. (2021) 5:116. doi: 10.1210/jendso/bvab116 PMC8294688 34308090

[86] Zhang H Ke Z Dong S du Y Tang W Chen M. Eldecalcitol prevents muscle loss by suppressing PI3K/AKT/FOXOs pathway in orchiectomized mice. Front Pharmacol. (2022) 13:1018480. doi: 10.3389/fphar.2022.1018480, PMID: 36386197 PMC9650589

[87] Lopez P Newton RU Taaffe DR Winters-Stone K Galvão DA Buffart LM. Moderators of resistance-based exercise programs' effect on sarcopenia-related measures in men with prostate cancer previously or currently undergoing androgen deprivation therapy: An individual patient data meta-analysis. J Geriatr Oncol. (2023) 14:101535. doi: 10.1016/j.jgo.2023.101535, PMID: 37229882

[88] Campbell KL Zadravec K Bland KA Chesley E Wolf F Janelsins MC. The effect of exercise on Cancer-related cognitive impairment and applications for physical therapy: systematic review of randomized controlled trials. Phys Ther. (2020) 100:523–42. doi: 10.1093/ptj/pzz090, PMID: 32065236 PMC8559683

[89] Ohtaka A Aoki H Nagata M Kanayama M Shimizu F Ide H. Sarcopenia is a poor prognostic factor of castration-resistant prostate cancer treated with docetaxel therapy. Prostate Int. (2019) 7:9–14. doi: 10.1016/j.prnil.2018.04.002, PMID: 30937292 PMC6424678

[90] Lee JH Jee BA Kim JH Bae H Chung JH Song W. Prognostic impact of sarcopenia in patients with metastatic hormone-sensitive prostate Cancer. Cancers. (2021) 13:6345. doi: 10.3390/cancers13246345, PMID: 34944964 PMC8699789

[91] Iwamoto H Kano H Shimada T Naito R Makino T Kadomoto S. Sarcopenia and visceral metastasis at Cabazitaxel initiation predict prognosis in patients with castration-resistant prostate Cancer receiving Cabazitaxel chemotherapy. In Vivo. (2021) 35:1703–9. doi: 10.21873/invivo.12430, PMID: 33910855 PMC8193302

[92] Couderc AL Villani P Berbis J Nouguerède E Rey D Rossi D. HoSAGE: sarcopenia in older patient with intermediate/high-risk prostate cancer, prevalence and incidence after androgen deprivation therapy: study protocol for a cohort trial. BMC Cancer. (2022) 22:78. doi: 10.1186/s12885-021-09105-8, PMID: 35042460 PMC8764762

[93] Ogasawara N Nakiri M Kurose H Ueda K Chikui K Nishihara K. Sarcopenia and excess visceral fat accumulation negatively affect early urinary function after I-125 low-dose-rate brachytherapy for localized prostate cancer. Int J Urol. (2023) 30:347–55. doi: 10.1111/iju.15120, PMID: 36520921

[94] Khan M Parshad S Naimi MF Sidhu AK Lyons F Hardisty MR. Sarcopenia in men with bone-predominant metastatic castration-resistant prostate Cancer undergoing Ra-223 therapy. Clin Genitourin Cancer. (2023) 21:e228–e235.e1. doi: 10.1016/j.clgc.2023.01.009, PMID: 36849325

[95] Hiroshige T Ogasawara N Kumagae H Ueda K Chikui K Uemura KI. Sarcopenia and the therapeutic effects of androgen receptor-axis-targeted therapies in patients with castration-resistant prostate Cancer. In Vivo. (2023) 37:1266–74. doi: 10.21873/invivo.13204, PMID: 37103069 PMC10188008

[96] Mitsui Y Araki M Maruyama Y Sadahira T Wada K Edamura K. 884 - sarcopenia patients are clinically dissatisfied with postoperative urinary function compared with non-sarcopenia patients in robot-assisted radical prostatectomy. Eur Urol Suppl. (2019) 18:e1194–5. doi: 10.1016/S1569-9056(19)30859-0

[97] Mitsui Y Sadahira T Maruyama Y Sato R Rodrigo AGH Wada K. Impact of sarcopenia on erectile function after nerve-sparing robot-assisted radical prostatectomy. World J Mens Health. (2021) 39:673–82. doi: 10.5534/wjmh.200036, PMID: 33474847 PMC8443993

[98] Mason RJ Boorjian SA Bhindi B Rangel L Frank I Karnes RJ. The association between sarcopenia and oncologic outcomes after radical prostatectomy. Clin Genitourin Cancer. (2018) 16:e629–36. doi: 10.1016/j.clgc.2017.11.003, PMID: 29289518

[99] Pak S Park SY Shin TJ You D Jeong IG Hong JH. Association of muscle mass with survival after radical prostatectomy in patients with prostate cancer. J Urol. (2019) 202:525–32. doi: 10.1097/JU.0000000000000249, PMID: 30916628

[100] Capitanio U Bensalah K Bex A Boorjian SA Bray F Coleman J. Epidemiology of renal cell carcinoma. Eur Urol. (2019) 75:74–84. doi: 10.1016/j.eururo.2018.08.036, PMID: 30243799 PMC8397918

[101] Yuxuan L Junchao L Wenya L. The role of sarcopenia in treatment-related outcomes in patients with renal cell carcinoma: a systematic review and meta-analysis. Medicine. (2022) 101:e31332. doi: 10.1097/MD.0000000000031332, PMID: 36316941 PMC9622586

[102] Hu X Liao DW Yang ZQ Yang WX Xiong SC Li X. Sarcopenia predicts prognosis of patients with renal cell carcinoma: a systematic review and meta-analysis. Int Braz J Urol. (2020) 46:705–15. doi: 10.1590/S1677-5538.IBJU.2019.0636, PMID: 32213202 PMC7822353

[103] Fukushima H Nakanishi Y Kataoka M Tobisu K Koga F. Prognostic significance of sarcopenia in patients with metastatic renal cell carcinoma. J Urol. (2016) 195:26–32. doi: 10.1016/j.juro.2015.08.071, PMID: 26292042

[104] Ueki H Hara T Okamura Y Bando Y Terakawa T Furukawa J. Association between sarcopenia based on psoas muscle index and the response to nivolumab in metastatic renal cell carcinoma: a retrospective study. Investig Clin Urol. (2022) 63:415–24. doi: 10.4111/icu.20220028, PMID: 35796138 PMC9262481

[105] Herrmann T Mione C Montoriol PF Molnar I Ginzac A Durando X. Body mass index, sarcopenia, and their variations in predicting outcomes for patients treated with Nivolumab for metastatic renal cell carcinoma. Oncology. (2022) 100:114–23. doi: 10.1159/000520833, PMID: 34999587

[106] Buchler T Kopecka M Zemankova A Wiesnerová M Streckova E Rozsypalova A. Sarcopenia in metastatic renal cell carcinoma patients treated with Cabozantinib. Target Oncol. (2020) 15:673–9. doi: 10.1007/s11523-020-00744-8, PMID: 32748047

[107] Colomba E Alves Costa Silva C Le Teuff G Elmawieh J Afonso D Benchimol-Zouari A. Weight and skeletal muscle loss with cabozantinib in metastatic renal cell carcinoma. J Cachexia Sarcopenia Muscle. (2022) 13:2405–16. doi: 10.1002/jcsm.13021, PMID: 35903892 PMC9530538

[108] Ishihara H Kondo T Omae K Takagi T Iizuka J Kobayashi H. Sarcopenia and the modified Glasgow prognostic score are significant predictors of survival among patients with metastatic renal cell carcinoma who are receiving first-line Sunitinib treatment. Target Oncol. (2016) 11:605–17. doi: 10.1007/s11523-016-0430-0, PMID: 27023922

[109] Antoun S Baracos VE Birdsell L Escudier B Sawyer MB. Low body mass index and sarcopenia associated with dose-limiting toxicity of sorafenib in patients with renal cell carcinoma. Ann Oncol. (2010) 21:1594–8. doi: 10.1093/annonc/mdp605, PMID: 20089558

[110] Huillard O Mir O Peyromaure M Tlemsani C Giroux J Boudou-Rouquette P. Sarcopenia and body mass index predict sunitinib-induced early dose-limiting toxicities in renal cancer patients. Br J Cancer. (2013) 108:1034–41. doi: 10.1038/bjc.2013.58, PMID: 23462722 PMC3619075

[111] Gu W Wu J Liu X Zhang H Shi G Zhu Y. Early skeletal muscle loss during target therapy is a prognostic biomarker in metastatic renal cell carcinoma patients. Sci Rep. (2017) 7:7587. doi: 10.1038/s41598-017-07955-6, PMID: 28790354 PMC5548774

[112] Otemuyiwa B Derstine BA Zhang P Wong SL Sabel MS Redman BG. Dorsal muscle attenuation May predict failure to respond to Interleukin-2 therapy in metastatic renal cell carcinoma. Acad Radiol. (2017) 24:1094–100. doi: 10.1016/j.acra.2017.03.003 28341412

[113] Lee J Suh J Song C You D Jeong IG Hong B. Association between sarcopenia and survival of patients with organ-confined renal cell carcinoma after radical nephrectomy. Ann Surg Oncol. (2022) 29:2473–9. doi: 10.1245/s10434-021-10881-7, PMID: 34625877

[114] Higgins MI Martini DJ Patil DH Nabavizadeh R Steele S Williams M. Sarcopenia and modified Glasgow prognostic score predict postsurgical outcomes in localized renal cell carcinoma. Cancer. (2021) 127:1974–83. doi: 10.1002/cncr.33462, PMID: 33760232

[115] Midenberg E Higgins MI Schmeusser BN Patil DH Zaldumbide J Martini DJ. Prognostic value of sarcopenia and albumin in the surgical Management of Localized Renal Cell Carcinoma. Urol Oncol. (2023) 41:50.e19–26. doi: 10.1016/j.urolonc.2022.09.020, PMID: 36280529

[116] Makino T Izumi K Iwamoto H Kadomoto S Mizokami A. Combination of sarcopenia and hypoalbuminemia is a poor prognostic factor in surgically treated nonmetastatic renal cell carcinoma. Biomedicines. (2023) 11:1604. doi: 10.3390/biomedicines11061604, PMID: 37371699 PMC10295698

[117] Sharma P Zargar-Shoshtari K Caracciolo JT Fishman M Poch MA Pow-Sang J. Sarcopenia as a predictor of overall survival after cytoreductive nephrectomy for metastatic renal cell carcinoma. Urol Oncol. (2015) 33:339.e17–23. doi: 10.1016/j.urolonc.2015.01.011, PMID: 26094169

[118] Khan AI Psutka SP Patil DH Hong G Williams MA Bilen MA. Sarcopenia and systemic inflammation are associated with decreased survival after cytoreductive nephrectomy for metastatic renal cell carcinoma. Cancer. (2022) 128:2073–84. doi: 10.1002/cncr.34174, PMID: 35285950

[119] Mao W Wang K Zhang H Lu H Sun S Tian C. Sarcopenia as a poor prognostic indicator for renal cell carcinoma patients undergoing nephrectomy in China: a multicenter study. Clin Transl Med. (2021) 11:e270. doi: 10.1002/ctm2.270, PMID: 33463055 PMC7775986

[120] Noguchi G Kawahara T Kobayashi K Tsutsumi S Ohtake S Osaka K. A lower psoas muscle volume was associated with a higher rate of recurrence in male clear cell renal cell carcinoma. PLoS One. (2020) 15:e0226581. doi: 10.1371/journal.pone.0226581, PMID: 31895931 PMC6939903

[121] Mao W Zhang N Wang K Hu Q Sun S Xu Z. Combination of albumin-globulin score and sarcopenia to predict prognosis in patients with renal cell carcinoma undergoing laparoscopic nephrectomy. Front Nutr. (2021) 8:731466. doi: 10.3389/fnut.2021.731466, PMID: 34631767 PMC8495413

[122] Schmeusser BN Biermann H Nicaise EH Ali AA Patil DH Midenberg E. Creatinine to cystatin-C ratio in renal cell carcinoma: a clinically pragmatic prognostic factor and sarcopenia biomarker. Oncologist. (2023) 28:e1219–29. doi: 10.1093/oncolo/oyad218, PMID: 37540787 PMC10712910

[123] Phuong A Marquardt JP O'Malley R Holt SK Laidlaw G Eagle Z. Changes in skeletal muscle and adipose tissue during cytotoxic chemotherapy for testicular germ cell carcinoma and associations with adverse events. Urol Oncol. (2022) 40:456.e19–30. doi: 10.1016/j.urolonc.2022.07.013, PMID: 36028450

[124] Baky F Howard JM Ashbrook C Jafri F Chertack N Woldu S. Decreased muscle mass prior to and following chemotherapy predicts morbidity in testicular Cancer patients undergoing post-chemotherapy retroperitoneal lymph node dissection. Clin Genitourin Cancer. (2022) 20:e460–4. doi: 10.1016/j.clgc.2022.06.007, PMID: 35798646

[125] Fraisse G Renard Y Lebacle C Masson-Lecomte A Desgrandchamps F Hennequin C. La sarcopénie est-elle un facteur de morbi-mortalité dans le traitement des tumeurs localisées de la vessie infiltrant le muscle? [Is sarcopenia a morbi-mortality factor in the treatment of localized muscle-invasive bladder cancer?]. Prog Urol. (2020) 30:41–50. doi: 10.1016/j.purol.2019.11.002, PMID: 31818689

